# Premature aging in genetic diseases: what conclusions can be drawn for physiological aging

**DOI:** 10.3389/fragi.2023.1327833

**Published:** 2024-02-28

**Authors:** Filip Milosic, Markus Hengstschläger, Selma Osmanagic-Myers

**Affiliations:** Center for Pathobiochemistry and Genetics, Medical University of Vienna, Vienna, Austria

**Keywords:** aging, premature aging, progeroid, senescence, cockayne syndrome, HGPS, werner syndrome

## Abstract

According to current views the major hallmarks of physiological aging may be subdivided into three categories, primary causes of cellular damage (genomic instability, telomere attrition, loss of proteostasis, epigenetic alterations and compromised macroautophagy), antagonistic hallmarks that represent response to damage (deregulated nutrient sensing, cellular senescence, mitochondrial dysfunction) and integrative hallmarks that represent culprits of the phenotype (stem cell exhaustion, altered intercellular communication, chronic inflammation, dysbiosis). In contrast to physiological aging, premature aging diseases are driven by one or two distinct primary causes of aging, such as genomic instability in the case of Werner syndrome (WS), each displaying other hallmarks of aging to a variable extent. In this review we will focus on primary causes of well-investigated premature aging diseases Hutchinson-Gilford progeria syndrome (HGPS), WS, and Cockayne syndrome (CS) and for each provide an overview of reported aging hallmarks to elucidate resemblance to physiological aging on the mechanistic level and in the context of characteristic age-related diseases. Ubiquitous and tissue specific animal models of premature aging diseases will be discussed as useful tools to decipher fundamental aging-related mechanisms and develop intervention strategies to combat premature aging and age-related diseases.

## Introduction

According to the most recent definition, aging is the process of molecular and cellular damage accumulation that leads to functional decline, chronic diseases, increased morbidity and mortality (Moqri et al., 2023). During the aging process different alterations are manifested, characterized as “aging hallmarks”, that may be subdivided according to current views into three categories, primary, antagonistic and integrative hallmarks. As postulated by Lopez-Otin and colleagues and explained in detail in (Lopez-Otin et al., 2013; Lopez-Otin et al., 2023) primary hallmarks are usually considered as primary causes of cellular damage. These include aging-induced changes to the genome, epigenome, telomeres, proteome and organelles that mainly occur due to genomic instability, epigenetic alterations, telomere attrition, loss of proteostasis and disabled macroautophagy, respectively. Antagonistic hallmarks arise as a response to counteract cellular damage and may have variable functions at different developmental stages. Integrative hallmarks characterize cumulative changes occurring if the damage caused by primary and antagonistic hallmarks cannot be compensated. These so called “culprits of the phenotype” will further perturb tissue homeostasis.

In this review we will first provide a short introduction to major progeroid syndromes including description and molecular basis of selected well-investigated premature aging diseases, Hutchinson-Gilford progeria syndrome (HGPS), Werner syndrome (WS), and Cockayne syndrome (CS). In the second and third part we aim to delineate commonalities and differences at the molecular, tissue and disease level of these progeroid disorders to physiological aging. In the last part we will elaborate on intervention strategies in progeroid syndromes and relate to those applicable to age-related diseases. In each part we will attempt to provide common mechanistic links applicable to physiological aging with an aim to extract lessons we learned from these premature aging disorders and an intention to pave ground for future research avenues.

### Progeroid syndromes

**FIGURE 1:**
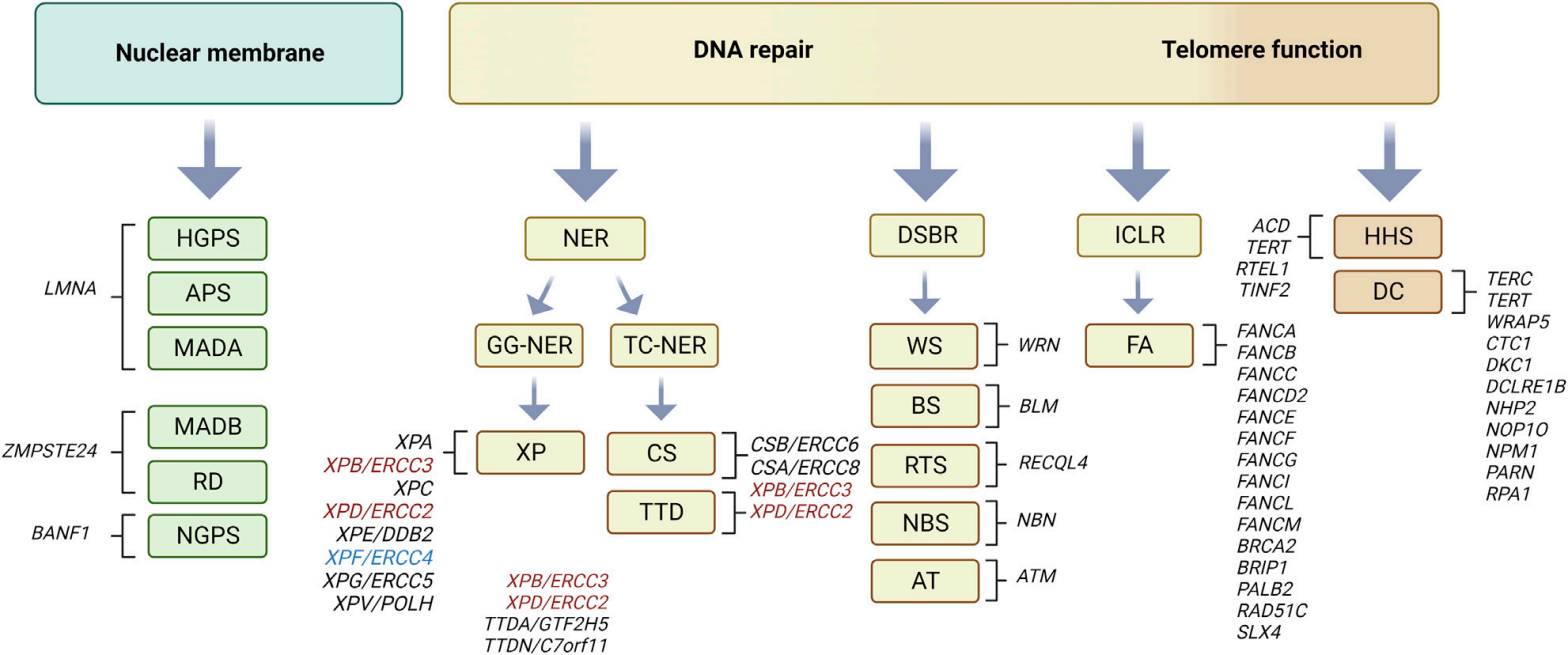
Progeroid syndromes classified according to affected molecular pathway. Nuclear membrane involvement: Hutchinson-Gilford progeria syndrome (HGPS), atypical progeria syndromes (APS), mandibuloacral dysplasia type A and B (MADA, MADB), restrictive dermopathy (RD), and Nestor-Guillermo progeria syndrome (NGPS) (reviewed in (Foo et al., 2019). DNA damage repair pathways involving nucleotide excision repair (NER): Cockayne syndrome (CS), xeroderma pigmentosum (XP), trichothiodystrophy (TTD) reviewed in (Niedernhofer et al., 2011). Note that dependent on mutations, ERCC3 (XPB) and ERCC2 (XPD) defects may lead to XP, CS or TTD phenotype (marked in red), whereas that in ERCC4 (XPF) to XP or FA (marked in blue) as reviewed in (Black, 2016). Double-strand break repair (DSB): Werner syndrome (WS), Rothmund-Thomson syndrome (RTS), Bloom syndrome (BS), Ataxia telangiectasia (ATM), Nijmegen breakage syndrome (NBS) and Fanconi anemia (FA) reviewed in (Carrero et al., 2016; Fiesco-Roa et al., 2022). Subgroup of latter involving telomere dynamics: dyskeratosis congenita (DC) and Hoyeraal-Hreidarsson syndrome (HHS) reviewed in (Savage, 2022). Affected genes in each of the progeroid syndromes are depicted in text next to corresponding brackets. Figure created with Biorender.com.

Progeroid syndromes show premature or accelerated features of aging in specific tissues with disease characteristics more or less accurately resembling corresponding age-related diseases in the elderly. According to previous reports (Carrero et al., 2016) the majority of progeroid syndromes may be subdivided into two categories depending on the molecular function of affected proteins. The first category comprises progeroid syndromes with gene mutations encoding proteins with important function for nuclear envelope stability and organization such as Hutchinson-Gilford progeria syndrome (HGPS), atypical progeria syndromes (APS), mandibuloacral dysplasia type A and B (MADA, MADB), restrictive dermopathy (RD) and Nestor-Guillermo progeria syndrome (NGPS). The components of the first category may be submerged according to affected genes to three groups: i) HGPS, APS and MADA with mutations in *LMNA* gene, ii) MADB and RD involving mutations in *ZMPSTE24* gene and iii) NGPS with mutations in *BANF1* gene (Figure 1; reviewed in (Foo et al., 2019)). The second category of progeroid syndromes comprises genes involved in DNA damage repair pathways such as trichothiodystrophy (TTD), xeroderma pigmentosum (XP), Cockayne syndrome (CS), Werner syndrome (WS), Bloom syndrome (BS), Rothmund-Thomson syndrome (RTS), Nijmegen breakage syndrome (NBS), Ataxia telangiectasia (AT) and Fanconi anemia (FA). According to types of DNA damage repair pathway affected, the second class of progeroid syndromes may be again further submerged into three categories: i) global genome (GG) and transcription-coupled (TC) nucleotide excision pathway (NER), (ii) double-strand break repair (DSBR) and iii) interstrand DNA crosslink link repair (ICLR) pathway (Figure 1; see Box 1; reviewed in (Carrero et al., 2016; Fiesco-Roa et al., 2022; Niedernhofer et al., 2011)). A subtype of the second category involves mutations in genes that belong to telomere biology disorders such as dyskeratosis congenita (DC) and Hoyeraal-Hreidarsson syndrome (HHS) (Savage, 2022). It must be noted that this is approximate type of classification since some affected proteins may be involved in multiple pathways such as in addition to DSBR, the enrollment of Werner helicase (WRN) in BER-pathway and telomere dynamics (Rossi et al., 2010), that of Ataxia telangiectasia mutated kinase (ATM) in NER-pathway (Ray et al., 2016). Furthermore, different mutations within the same XP genes may lead to different clinical phenotypes (Kraemer et al., 2007; Black, 2016). The classification of the listed progeroid syndromes according to prevalent molecular function of affected proteins is summarized in Figure 1. Most progeroid syndromes develop general premature aging features such as hair graying and hair loss, age-related changes of the skin and short stature in conjunction with specific age-related disease characteristics and clinical phenotypes reviewed in (Schnabel et al., 2021).

Box 1. **Explanation of basic terminologies and key components relevant for the text.**

**Base excision repair (BER)**: a type of single-strand DNA break (SSB) repair of small oxidative, deaminated and alkylated DNA lesions that generally introduce no or minimal DNA helix distortions. Key components engaged in BER are glycosylase (e.g., NEIL) that removes damaged DNA base creating apurinic (AP) site, AP endonuclease to remove AP site creating SSB, Polymerase β to incorporate correct nucleotides and DNA-flap endonuclease (e.g., FEN1) to remove generated single-stranded DNA flaps that may form (reviewed in (Gohil et al., 2023).

**Nucleotide excision repair (NER)**: a type of single-strand DNA break (SSB) repair that removes bulky helix-distorting lesions such as cyclobutane pyrimidine dimers. The latter can be recognized throughout the whole genome (GG-NER) or specifically on transcribing strands of active genes (TC-NER). Helix distortions in GG-NER are recognized by Xeroderma pigmentosum complementation group protein C, XPC, whereas in TC-NER by the CS group CSB and CSA (details in text). After damage recognition common mechanisms in TC-NER and GG-NER are used to excise the DNA damaged sites (reviewed in detail in (de Boer and Hoeijmakers, 2000; Kraemer et al., 2007; Niedernhofer, 2008).

**Double-strand break repair (DSBR)**: operates to repair double-strand DNA breaks, usually occurring after collapse of replication forks, either through non-homologous end-joining (NHEJ) or in S and G2 phases of the cell cycle, when sister chromatids can be used as templates, through homologous recombination (HR). Some basic NHEJ components are damage recognition KU proteins, DNA dependent protein kinase (DNA-PK) complex necessary for phosphorylation of histone H2AX and DNA ligases that perform relegation such as (LIG4). In HR, MRN (MRE11-RAD50-NBS) complex protein plays an important role in DSB recognition, nick generation and together with exonuclease 1 (EXO1) and/or nuclease DNA2 5′end-resection creating single stranded DNA (ssDNA) overhangs. Rad52 facilitates the loading of Rad51 forming nucleofilaments along ssDNA, strand invasion and annealing with matching sequences in the undamaged sister chromatids to form a displacement D-loop. The extension of the invading strand is mediated by DNA polymerase δ (Pol δ). Finally, D-loops may be resolved by migrating Holiday junctions through resolvases or BLM-RecQ-RMI-toposiomerase 3a (TOP3a) complex (reviewed in (Panier and Boulton, 2014).

**Interstrand DNA crosslink repair (ICLR)**: is used to repair covalent bonds between opposite DNA strands. Throughout the cell cycle proteins involved in TC-NER or GG-NER may be utilized in ICLR to repair the damage. In the S phase of the cell cycle ICLR engages HR components but also additionally factors responsible for Fanconi anemia such as proteins encoded by *FANC* genes (Figure 1; reviewed in (Hashimoto et al., 2016; Fiesco-Roa et al., 2022).

**Telomeres, telomerase and shelterin complex**: Telomeres are tandem repeats of TTAGGG at the end of linear chromosomes that cannot be synthetized by classic DNA polymerase but require a specific telomerase reverse transcriptase (*TERT* gene) that utilizes telomerase RNA (*TERC* gene) as a template. Dyskerin (*DKC1* gene), TCAB1 (*WRAP53* gene) and others are essential components of the telomerase complex. Shelterin complex associates with telomeres and plays a crucial role in telomere length maintenance by protecting them from being recognized as DNA damage sites. Six protein subunits TRF1, TRF2, TIN2, hRap1, TPP1 and POT1 are parts of shelterin complex. In addition, regulator of telomere elongation helicase 1 (*RTEL1*) confers t-loop stability playing an important role in telomere replication. See details on genes and telomere biology disorders (Martinez and Blasco, 2017; Savage, 2022).

***BANF1*** *:* gene encoding for the protein barrier to autointegration factor 1 involved in chromatin and nuclear organization.

**XP group of proteins**: comprise seven complementation group members (A, B, C, D, E, F, G) important for repair of damage particularly upon exposure to UV light through NER (reviewed in (Niedernhofer, 2008; Niedernhofer et al., 2011).

**Z*MPSTE24***: gene encoding a Zinc metalloprotease related to the STE24 homology in yeast (alternative name: FACE1) involved in processing of prelamin A to mature lamin A.

### Introduction to selected progeroid syndromes HGPS, WS and CS

In this part we will give a brief introduction providing molecular basis of selected well-investigated progeriod syndromes, one from the first above-described category, Hutchinson-Gilford progeria syndrome (HGPS), and two from the second category, Werner syndrome (WS) and Cockayne syndrome (CS). Particularly intriguing we find comparison of HGPS and WS that despite entirely different molecular functions of affected proteins develop some similar clinical features such as accelerated cardiovascular disease (CVD) and atherosclerosis. On the other hand, why do CS patients, despite having deficits in repair of DNA damage similar to WS, develop entirely different clinical features involving neurodegeneration? We will address these and similar aspects particularly in the last parts of the manuscript where we describe in detail the disease pathology of these progeroid syndromes.

### Hutchinson-Gilford progeria syndrome

Hutchinson-Gilford progeria syndrome (HGPS) is an autosomal dominant extremely rare (1 in ∼4 million live births) progeroid disease with typical premature aging features such as short stature, skin scleroderma, alopecia, joint contractures, lipodystrophy, osteolysis, and most striking accelerated cardiovascular disease accompanied by severe atherosclerosis development (Merideth et al., 2008; Olive et al., 2010; Gordon et al., 2012). HGPS patients experience increased incidence of strokes and if not treated die in their teens at the age of ∼14 years due to myocardial infarction (Merideth et al., 2008; Silvera et al., 2013). HGPS as well as APS and MADA are caused by mutations in the *LMNA* gene that together with MADB, RD, and NGPS belong to a group of progeroid syndromes associated with defects in nuclear organization and stability (Foo et al., 2019). *LMNA* gene encodes for the lamin A protein and an alternative splice variant lamin C, which together with lamins B1 and B2 constitute parts of a dense intermediate filament meshwork (type V) just beneath the inner nuclear membrane, the so called nuclear lamina (Buxboim et al., 2023). Lamins are key nucleocytoskeletal connectors conferring shape and stability to the nucleus (Osmanagic-Myers et al., 2015; Buxboim et al., 2023). The role of lamins goes beyond a solely mechanical function and includes a plethothora of other tasks such as heterochromatin organization, scaffolding of transcriptional factors and affecting DNA replication and DNA repair as well (Dechat et al., 2010; Gonzalo, 2014; Osmanagic-Myers and Foisner, 2019).

In the majority of cases, HGPS is caused by *de novo* single-base substitution G608G (GGC>GGT) in exon 11 of *LMNA* gene with no change in the coding amino acid (De Sandre-Giovannoli et al., 2003; Eriksson et al., 2003). Lamin A, expressed as prelamin A, undergoes extensive post-translational modifications including farnesylation, carboxymethylation and finally cleavage of carboxyl terminal 15 amino acids by Zmpste24/FACE1 that results in removal of these post-translational modifications and formation of mature lamin A (Dechat et al., 2010). HGPS mutation leads to activation of cryptic RNA splice donor site within exon 11 of *LMNA* gene and formation of shorter, permanently farnesylated prelamin A named progerin (Gordon et al., 2012). Permanently farnesylated prelamin A is toxic to cells inducing nuclear shape irregularities (De Sandre-Giovannoli et al., 2003; Eriksson et al., 2003) such as lobulation of the nuclear envelope, thickening of the lamina, loss of peripheral heterochromatin, clustering of nuclear pore complexes and linkers of nucleoskeleton and cytoskeleton (LINC) Sun proteins (Goldman et al., 2004; Chen et al., 2012). Similar effects are observed in MADA, MADB, and RD associated with accumulation of prelamin A either due to mutations in different parts of *LMNA* gene or those affecting *ZMPSTE24* gene that lead to total or partial loss of its proteolytic activity (Figure 1 (Foo et al., 2019)). Treatment of cells with farnesyltransferase inhibitor lonafarnib or antisense oligonucleotides to target mutated splice site restores nuclear architecture and rescues nuclear defects (Capell et al., 2005; Scaffidi and Misteli, 2005). In addition, as expected from multifaceted role of lamin A, it is conceivable that many other lamin A functions are perturbed by accumulation of mutated prelamin A as depicted in more details in the following chapters.

### Werner syndrome

Werner syndrome (WS) is an autosomal recessive disorder characterized by progeroid features that develop at late adolescence such as short stature, atrophied skin, loss of subcutaneous fat, graying of the hair, cataracts, type 2 diabetes mellitus (T2DM), osteoporosis, atherosclerosis and increased incidence of cancer (Oshima et al., 2017). Classical WS is caused by mutations in the *WRN* gene encoding a WRN-helicase that belongs to the family of five RecQ-helicases: RecQ1, WRN, BLM, RecQ4 and RecQ5. Mutations in three of these RecQ family members, *WRN*, *BLM*, *RecQ4* lead to *WS*, *BS* and *RTS* (Figure 1), respectively, whereby WS is considered a prototype progeroid disorder (de Renty and Ellis, 2017). WRN helicase, is an ATP-dependent 5′-3′ DNA unwinding enzyme harboring also 3′-5′ exonuclease activity. It has a key role in preserving genome stability through several important functions in DNA replication, telomere maintenance, and DNA damage repair (Rossi et al., 2010; Shimamoto et al., 2015). Both helicase and exonuclease activities are essential for resolving DNA forks, D-loops, Holliday junctions and telomeric G-quadruplexes (G4) that normally occur during DNA replication but also at sites of DNA damage (Rossi et al., 2010). One of its key roles is enabling restart of stalled replication forks that occur at sites of DNA damage or G-quadruplexes formed at G-rich telomere sequences (Kipling et al., 2004; Shimamoto et al., 2015). WRN promotes DNA damage repair pathway also through interaction with several major players involved in base excision repair (BER) and double-strand break repair (DSBR; see Box 1). In BER, WRN interacts with glycosylase NEIL1 promoting removal of DNA lesions by this enzyme (Das et al., 2007). Through direct binding and helicase activity WRN stimulates the strand displacement polymerase β DNA synthesis (Harrigan et al., 2003) and enhances the efficiency of exonuclease FEN1 mediating removal of generated DNA flaps (Brosh et al., 2002). In DSBR involving non-homologous end-joining (NHEJ, see Box 1), WRN binds to Ku-proteins in the presence of which its additional intrinsic exonuclease activity is strongly enhanced that contributes to efficient digestion of DNA lesions at DSB termini (Cooper et al., 2000). In parallel, WRN physically interacts with DNA-PK which is suggested to structurally stabilize this complex. Consistent with this role, in the absence of WRN, reduced rate of NHEJ associated with slower loss of γH2AX foci is observed (Grundy et al., 2016). In HR-repair pathway WRN helicase binding to MRE11 and NBS components of MRN complex and nuclease DNA2 promotes 5′ end-resection and generation of single strand DNA overhangs (Cheng et al., 2004; Lu and Davis, 2021). Furthermore, WRN binding to Rad52 increases the efficiency of Rad52-mediated annealing of the invading strand. Thereby, WRN presumably promotes double-strand opening in homologous DNA strand through its helicase activity (Baynton et al., 2003). WRN is further implicated in the process of translocation of Holliday junctions (*WRN* functions in detail reviewed in (Lu and Davis, 2021).

### Cockayne syndrome

Cockayne syndrome (CS) together with trichothiodystrophy (TTD) and xeroderma pigmentosum (XP), belongs to the group of nucleotide excision repair (NER) progeroid disorders (Figure 1) that affect transcription coupled NER (TC-NER) and global genome NER (GG-NER), respectively (Niedernhofer et al., 2011). CS is in ∼90% of cases caused by mutations in the “excision repair cross complementation group” (*ERCC*) genes 8 and 6, encoding Cockayne syndrome A (CSA) and B (CSB) proteins, respectively. The majority of mutations reside in the *ERCC6* gene (CSB) with many being compound heterozygous (Jaarsma et al., 2013). CSB protein acts as SNF family chromatin remodeler with ATPase activity that together with CSA protein is best known for the role in transcription-coupled nucleotide-excision DNA repair (TC-NER). These proteins also have a role in repair of DSB, and a variety of other functions as well (Jaarsma et al., 2013; Vessoni et al., 2020; Walker and Zhu, 2022). Mechanistically, during TC-NER, CSB recognizes nuclear but also mitochondrial DNA lesions (Scheibye-Knudsen et al., 2012; Scheibye-Knudsen et al., 2013) that cause RNA stalling in the active transcribed regions, and in turn promotes either backtracking of stalled RNA polymerase or its removal via ubiquitination. The latter is mediated through recruitment of CSA and associated E3 ubiquitin ligase complex. After initial DNA damage recognition, TC-NER and GG-NER use common mechanisms to remove DNA lesions (Kraemer et al., 2007; Niedernhofer, 2008). This involves recruitment of a 10 protein complex of the basal transcription factor that is also part of NER, TFIIH with helicases XPB and XPD that unwind the DNA followed by incision/excision of damaged DNA via XPF-ERCC1 and XPG complexes, and finally gap filling through DNA replication and ligation (Kraemer et al., 2007; Paccosi et al., 2022). Thus, since GG-NER and TC-NER converge at later stages of the NER, it is not surprising that different XP mutations in the same genes involved in later stages of the NER may develop to TC-NER disorders (Kraemer et al., 2007) as depicted in Figure 1.

CS originally described in 1936 (Cockayne, 1936) is inherited in an autosomal recessive fashion and has varying degrees of clinical symptoms ranging from moderate to severe, with average life expectancy of ∼16 and ∼5 years in type I and II CS, respectively. Type III CS displays a milder phenotype with average life expectancy of ∼30 years (Laugel, 2013; Vessoni et al., 2020). CS is characterized by typical short stature involving cachectic dwarfism, loss of subcutaneous fat, premature aging facial features, microcephaly, cerebellar ataxia due to strong effects in the neural system characterized by intellectual disability. It is mainly associated with severe white matter loss and atrophy of cerebrum, cerebellum and brain stem, pyramidal and extrapyramidal signs, peripheral and sensorial nerve impairment (hearing loss, retinopathy, cataracts) (Laugel, 2013; Karikkineth et al., 2017; Spitz et al., 2021). However, CS affects also other systems such as cardiovascular system with early onset hypertension, increased vasculature in subarachnoid space and subdural hematoma (Hayashi et al., 2012). In contrast to mutations in genes responsible involving GG-NER, CS is not associated with higher incidents of cancer (Lu et al., 2001).

## Hallmarks of aging in the context of progeroid syndromes

Here we will focus on aging hallmarks of selected progeroid syndromes HGPS, WS and CS and at relevant parts involve findings in other progeroid disorders as well. Mechanistic insights will be further elaborated particularly to those aspects hitherto relevant to physiological aging and key intervention strategies in progeroid syndromes. The emerging concept highlighting interconnection of aging hallmarks (Lopez-Otin et al., 2023) may provide explanation for the presence of almost all aging hallmarks in selected progeroid diseases as depicted below, even in the absence of obvious common disease-causing genes as in the case of WS and HGPS.

### Primary causes of damage in WS, CS and other DNA damage repair progeroid disorders

**FIGURE 2:**
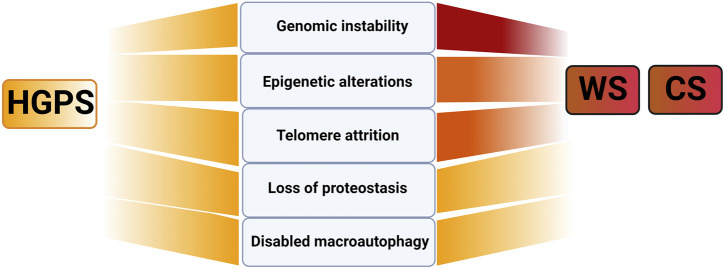
Primary aging hallmarks in selected progeroid disorders CS, WS and HGPS. Note that darker red shading indicates genomic instability together with epigenetic changes and telomere attrition as the primary cause of damage in WS and CS and uniform color in HGPS, no prevalent primary cause of damage in HGPS. Figure created with Biorender.com.

In regard to primary aging hallmarks involving causes of damage, it is difficult and probably incorrect to attribute to each of the here described progeroid syndromes one primary cause of damage. However, we will attempt to highlight those that are prevalent or appear most upstream causes of damage. For WS including its family members, BS and RTS (Rossi et al., 2010) but also for CS (Lodato et al., 2018) and XP (de Boer and Hoeijmakers, 2000) genomic instability, defined as the tendency of the genome to acquire mutations, appears to be the prevalent cause of damage (Figure 2). This is expected as exemplified in case of WS and CS due to reduced efficiency of DNA-repair machinery in the absence of functioning WRN-helicase, as a key protein involved in BER, NHEJ and HR and that of CS-proteins in TC-NER. Consistently, elevated levels of DNA damage “foci”, marked by ataxia ATM-phosphorylated histone 2A variant (γH2AX) and p53-binding protein 1 (53BP1) were reported for WS (Chang et al., 2004; Szekely et al., 2005; Saha et al., 2014; Zhang et al., 2015) and CS (Batenburg et al., 2015; Pascucci et al., 2018; Wang et al., 2020). Thereby is ATM the key kinase that recognizes these damaged DNA sites leading either directly or indirectly to phosphorylation of several downstream substrates such as NBS, Chk2/p53, γH2AX (Shiloh, 2003). Thus, it is not surprising that genomic instability is a prevalent feature in progeroid disorders affecting *ATM* gene (AT) and its family members (Figure 1) (Shiloh, 2003).

Another driving cause of damage in WS (Crabbe et al., 2004) and CS (Batenburg et al., 2012) is accelerated loss of telomeres (Figure 2). In physiological aging telomere attrition occurs due to inability of classical DNA polymerase to synthetize telomeric repeats at chromosome ends that cannot be entirely compensated by telomerase reverse transcriptase (Martinez and Blasco, 2017). In WS accelerated telomeric loss is linked to the key function of WS helicase in efficient replication process of G-rich telomeric DNA regions. Consequently, in mice with longer telomeres than humans, WS-like disease pathology could only be recapitulated upon breeding in mTERC−/− background (see Box1) highlighting telomere attrition as one the key causes of damage in WS (Chang et al., 2004). In CS, CSB association with shelterin protein complex subunit, a telomeric repeat binding factor 2 (TRF2), was shown to be essential for telomere protection that suppressed the formation of telomere-dysfunction induced foci (Batenburg et al., 2012). Thus, for both WS and CS elevated telomere attrition is expected to further exacerbate genomic instability by activating DNA-damage response and promoting chromosome fusions. This will add up to already elevated DNA damage in these progeroid syndromes which exemplifies the interconnectedness of aging hallmarks as explained in detail below (Crabbe et al., 2007; Rossi et al., 2010). Consistent with this notion genomic instability is also a prevalent cause of damage in typical telomere biology disorders with substantial telomere shortening, such as dyskeratosis congenita (DC) and Hoyeraal-Hreidarsson syndrome (HHS) (Figure 1) as reviewed in (Martinez and Blasco, 2017; Fiesco-Roa et al., 2022). Finally, genomic instability appears to be the driving cause of damage in progeroid disorders with defective ICL-repair (see Figure 1; ∼20 genes affected). Here due to lack of DSB repair through HR, cells entirely rely on error-prone NHEJ to repair the DNA damage resulting in severe cases of chromosome instabilities and cancer (Fiesco-Roa et al., 2022). Altogether, the above mentioned mechanisms may be highly relevant in deciphering major players and conditions causing decline in the efficiency of DNA repair machinery in physiological aging and age-related diseases (Gorbunova et al., 2007).

### Primary causes of damage in HGPS and other nuclear envelope disorders

For HGPS, several reports have validated increased levels of DNA damage (Liu et al., 2005; Varela et al., 2005; Osorio et al., 2011; Chojnowski et al., 2020) associated with replication stalling (Kreienkamp et al., 2018) and shift towards error prone NHEJ with reduced fidelity (Joudeh et al., 2023). Altogether all these factors contribute to increased genomic instability of progerin expressing cells (reviewed in (Gonzalo and Kreienkamp, 2015)). However, for HGPS no prevalent primary hallmark may be attributed since in addition to genomic instability (Liu et al., 2005; Liu et al., 2006), telomere attrition (Cao et al., 2011a), loss of proteostasis (see below; (Vidak et al., 2023)) and changes in epigenome appear to be similarly abundant as well ((Chojnowski et al., 2020) (Figure 2; Table 1). This may be explained by highly multifunctional role of lamins in diverse cellular function as stated above (Dechat et al., 2010; Buxboim et al., 2023). For similar reasons no dominant cause may be attributed to other nuclear envelope progeroid syndromes affecting *LMNA* and *ZMPSTE* genes as reviewed in (Gonzalo and Kreienkamp, 2015; Cenni et al., 2018).

**TABLE 1:** Different aging hallmarks for progeroid diseases HGPS, WS, and CS. Tissue and cell origins used in experiments are highlighted in the columns right to respective disease since they may contribute to variability. Primary vs. immortalized cell cultures are distinguished.

Hallmark/Disease	HGPS	References	WS	References	CS	References
Genomic instability	✓HGPS FB	Liu et al. (2005), Liu et al. (2006)Reviewed in Gonzalo and Kreienkamp (2015)	✓WS whole blood	Kyoizumi et al. (1998), Chang et al. (2004), Crabbe et al. (2007)Reviewed in Rossi et al. (2010)	✓CS patient FB & iFB	Pascucci et al. (2018)
			✓WS FB		✓CS neuronal tissue	Lodato et al. (2018)
					✓CS PFC single neurons	Kim et al. (2022)
Epigenetic alterations	✓HGPS FB	Shumaker et al. (2006), Heyn et al. (2013), Horvath et al. (2018), Chojnowski et al. (2020), Kohler et al. (2020)	✓WS whole blood	Heyn et al. (2013), Guastafierro et al. (2017); Maierhofer et al. (2019)	✓CS FB	Crochemore et al. (2023)
			✓WS LCL			
	✓HGPS LCL		✓WS MSC	Zhang et al. (2015)	CS3BE and CS1AN	Lee et al. (2019)
Telomere attrition	✓ HGPS FB	Cao et al. (2011a), Aguado et al. (2019)	✓WS FB	Crabbe et al. (2004), Crabbe et al. (2007), Gatinois et al. (2019)	✓CS FB	Batenburg et al. (2012)
	✓HGPS FB and iFB		✓WS iPSC			
Loss of proteostasis	✓HGPS 3T3L1 iFB (GFP-progerin), HGPS iFB and FB	Mateos et al. (2015)	✓WS FB and iFB	Maity et al. (2018)	✓CS iFB	Alupei et al. (2018); Qiang et al. (2021)
	✓HGPS FB	Vidak et al. (2023)				
		Cao et al. (2011b)				
Disabled macroautophagy	✓Zmste24−/− mice	Marino et al. (2008), Monterrubio-Ledezma et al. (2023)	✓*WS FB,* Wrn-1 KD C.e. & D	(Maity et al., 2018; Fang et al., 2019)	✓*Csb* ^*m/m*^ mice & MDF	Scheibye-Knudsen et al. (2012), Majora et al. (2018)
	✓HGPS FB*					
Deregulated nutrient sensing	✓Zmpst24 −/−MDSPC	Marino et al. (2008), Kawakami et al. (2019)	✓WS SVF	Sawada et al. (2023)	✓CS m/m mouse liver	Schumacher et al. (2008)
	✓Zmpst24 −/−skeletal muscle				✓CS iPSC-derived neural cells	Vessoni et al. (2016)
Cellular senescence	✓HGPS FB	Bramwell and Harries (2023)	✓WS FB	Bramwell and Harries (2023)	✓CS FB	Bramwell and Harries (2023)
	✓HGPS ECs	Cao et al. (2011a), Osorio et al. (2011), Atchison et al. (2020), Manakanatas et al. (2022), Xu et al. (2022)	✓WS SVF	Sawada et al. (2023)		
	✓HGPS iECs				inMSCs& inNSCs	Wang et al. (2020)
	✓HGPS FB					
	✓HGPS LAKI mice					
Mitochondrial dysfunction	✓Lmna^G609/G609^ mice	Rivera-Torres et al. (2013)	✓WS FB*,* Wrn-1 KD C.e. & D	Fang et al. (2019)	✓CS FB & IFB	Chatre et al. (2015)
	✓HGPS FB	Viteri et al. (2010)			✓*Csb* ^*m/m*^ mice & MDF	Scheibye-Knudsen et al. (2012)
	✓HGPS 3 3T3L1	Mateos et al. (2015)				
Altered intercellular communication	✓HGPS ECs and EC HGPS mice with other mouse models reviewed in Benedicto et al.,	Osmanagic-Myers et al. (2019), Sun et al. (2020), Benedicto et al. (2021), Manakanatas et al. (2022)	✓WS SVF	Sawada et al. (2023)	✓Csa−/− mice	Kajitani et al. (2021)
					✓Csa−/−Xpa−/− mice	
Chronic inflammation	✓HGPS ECs and HGPS EC mice with other mouse models reviewed in Benedicto et al.,	Osmanagic-Myers et al. (2019), Sun et al. (2020), Benedicto et al. (2021), Manakanatas et al. (2022)	✓WS SVF	Sawada et al. (2023)	✓Csa−/− mice	Kajitani et al. (2021)
					✓Csa−/−Xpa−/− mice	
Stem cell exhaustion	✓HGPS iMSCs	Scaffidi and Misteli (2008), Rosengardten et al. (2011), Choi et al. (2018), Kawakami et al. (2019)	✓WS SC SVF	Sawada et al. (2023)	inMSCs & inNSCs	Wang et al. (2022)
	✓HGPS inMSCs					
	✓skin HGPS mice		✓WS FB*,* Wrn-1 KD C.e. & D	Fang et al. (2019)		
	✓Zmpst24 −/−MDSPC					
Dysbiosis	*Lmna* ^*G609G/G609G*^ mice	Barcena et al. (2019)	X	X	X	X

^a^ Asterisk indicates alterations occurring in opposite directions compared to physiological aging, e.g., autophagy increase in HGPS, cells; EC, endothelial cell; EC HGPS mice, endothelial specific HGPS, mice; FB, primary fibroblasts; iFB, immortalized fibroblasts; MSCs, mesenchymal stem cells; iMSCs, immortalized MSCs; inMSCs and inNSCs, MSCs, and NSCs, differentiated from iPSCs of patients, respectively; LCL, immortalized B-cells (lymphoblastoid cell line; LCL); MDF, mouse dermal fibroblasts; MDSPC, muscle-derived stem cell/progenitor cells; NSCs, neural stem cells; 3 T3L1 pre-adipocyte cell line; PFC, prefrontal cortex; SC, stem cells; SVF, stromal vascular fraction from patient adipose tissue; *Wrn-1 KD* C.e. & D, Wrn-1, knockdown in *C. elegans* and *Drosophila*.

Some of the mechanisms underlying genomic instability in HGPS may be caused by changes in the lamina that perturb binding of factors involved in DNA damage repair. In such a way, delayed recruitment to DNA damage foci was shown in HGPS for phospho-NBS and MRE11 (Constantinescu et al., 2010), RAD51 and 53BP1 (Liu et al., 2005) which are mainly involved in HR and NHEJ, respectively (see Box 1). In analogy, it may be speculated that changes in lamina and lamina associated proteins known to be occurring under specific conditions during physiological aging (Swift et al., 2013; Kristiani et al., 2020; Kirkland et al., 2023) may contribute to age-induced changes in the recruitment of DNA damage repair (DDR) factors. For instance, recent findings show for lamin B association with 53BP1 and its release upon DDR. Authors suggested that lamin B may serve as a reservoir for 53BP1 allowing its efficient recruitment to DNA damage foci upon DDR signals, a process shown to be sensitive to alterations in lamin B levels (Etourneaud et al., 2021). Thus, it remains to be elucidated if similar regulatory mechanisms associated with structural changes in lamin A and DDR factor perturbations in HGPS may give us important clues for physiological aging.

### Epigenetic alterations

Epigenetic changes during aging affect posttranslational modifications of histones such as lysine methylations, cytosine 5-methylations at DNA regions within CpG-rich islands, chromatin remodelers and non-coding RNAs (Zhang et al., 2020). Repressive histone marks that involve lysine methylations of histones, usually associated with a more condensed state of chromatin, heterochromatin, reduce access to transcriptional factors causing gene silencing. Some of the well-known repressive histone marks involving lysine trimethylations (me3) on core histones 3 (H3) and 4 (H4) are H3K9me3 and H3K27me3. Typical active histone marks involve acetylations of lysine residues on histones such as H3K9ac and H3K27ac or specific methylations such as H3K4me3. In physiological aging a general loss of heterochromatin occurs, but this is a highly heterogeneous phenomenon in regard to different gene loci, cell context and state of the cell. For instance, in senescent cells, despite general heterochromatin loss, increase in particular senescence-associated heterochromatic foci (SAHF) involved in silencing of genes responsible for cell division is observed (Lee et al., 2020). Despite this heterogeneity there seems to be a global trend in reduction of core histone levels, and repressive histone marks such as H3K9me3 and H3K27me3 in physiological aging and progeroid syndromes (Booth and Brunet, 2016; Lee et al., 2020; Zhang et al., 2020).

Heterochromatin loss in HGPS appears to be rooted in downregulation of chromatin modifier, methyltransferase EZH2 and chromatin remodeler, heterochromatin protein 1 alpha (HP1a) (Goldman et al., 2004; Scaffidi and Misteli, 2005; Shumaker et al., 2006; Chojnowski et al., 2020). This may be caused by altered scaffolding function of lamin A in HGPS, alterations of which, may similarly contribute to delocalization and decreased levels of chromatin remodelers in physiological aging (Lee et al., 2020).

Through association with the key chromatin remodeler HP1a, H3K9me3-specific methyltransferase SUV39H1 and lamina-heterochromatin anchoring protein LAP2β, WRN appears to act as a gatekeeper of heterochromatin stability (Zhang et al., 2015). Consequently, global heterochromatin loss occurs in WS adding epigenetic modifications to other two dominant causes of damage in this disease (Figure 2). Importantly, since WRN, HP1a, and SUV39H1 decline was detected in cells from aged donors, similar mechanism may apply to physiological aging as well (Zhang et al., 2015).

Epigenetic alterations associated with global heterochromatin loss appear to dominate CS as well (Figure 2). Decondensed H3K9me3 loci were shown to be associated with excessive PARP activation and downregulation of H3K9me3-specific methyltransferases, SUV39H1 and SETDB1. These effects may be attributed to the key function of ATP-dependent chromatin remodeler CSB in actively controlling the packaging state of DNA thereby regulating protein access (Lee et al., 2019). Since CSB and SETDB1 levels were found to decline in cells from aged individuals and senescent cells similar mechanisms may operate in physiological aging as well (Crochemore et al., 2019; Lee et al., 2019).

### Interconnectedness of primary hallmarks: genomic instability and epigenetic changes

To demonstrate the causal complexity between aging hallmarks in progeroid syndromes relevant to physiological aging let us consider connections between the above highlighted genomic instability, and changes in the epigenetic landscape. A question that arises is how could increase in DNA damage be connected to global changes in the epigenetic landscape and altered chromatin accessibility observed in these progeroid models (Shumaker et al., 2006; Carrero et al., 2016; Chojnowski et al., 2020; Kohler et al., 2020). Besides direct enrollment of the affected proteins, the plausible explanation for the correlation of such phenomena is offered by recent findings showing that acute DNA damage erodes the epigenetic landscape presumably due to persistent relocation of chromatin modifiers (RCM) such as silent information regulators (sirtuins) or Ten-eleven translocation enzymes (TET) to sites of DNA damage, postulated as “Information Theory of Aging” (Yang et al., 2023). Consistent with this theory in progeroid syndromes where DNA damage is associated with excessive PARP activation, treatments improving the activity of sirtuins have shown to be very beneficial (Liu et al., 2012; Fang et al., 2014). To add to the complexity level of this scenario, in HGPS, that harbors wide-spread loss of heterochromatin (Shumaker et al., 2006; Chojnowski et al., 2020), progerin-induced perturbation of heterochromatic lamina-associated domains (LADs) may additionally contribute to changes in epigenetic landscape (Osmanagic-Myers and Foisner, 2019).

Moreover, predictable epigenetic changes involving DNA hypo-and hypermethylation (DNAm) particularly at CpG rich regions have even led to generation of epigenetics clocks licensed for estimating chronological age and used as biomarkers of biological aging (Horvath, 2013; Moqri et al., 2023). In analogy, all progeroid syndromes have been shown to exhibit DNAm changes resembling those observed in physiological aging to a variable extent (Guastafierro et al., 2017; Maierhofer et al., 2019; Crochemore et al., 2023). Using modified epigenetic clocks adopted to specific cell types, Horvath and colleagues could even validate epigenetic clocks showing age acceleration in HGPS (Horvath et al., 2018) further supporting the view that there is much more of tale to tell from studying progeroid disorders.

### Loss of proteostasis and disabled macroautophagy

The interconnectedness of primary aging hallmarks in the context of progeroid disorders can be further extended to last two primary hallmarks, loss of proteostasis and disabled macroautophagy. These are regulated through amounts of respective waste products generated, misfolded protein and dysfunctional organelles and the cells ability to get rid of the “excessive garbage material”. Waste products arise in respective progeroid syndromes due to accumulation of misfolded proteins involving accumulation of mutated proteins as well. The latter is mainly due to reduced efficiency and errors in DNA repair machinery that give rise to accumulations of mutations (Rossi et al., 2010; Lodato et al., 2018). In addition, CSB proteins directly stimulate RNA polymerase I transcription enhancing ribosome translational fidelity, a process that is impaired in CS (Alupei et al., 2018). Since the efficiency of the DNA damage repair machinery declines in physiological aging (Gorbunova et al., 2007), defects in similar processes may contribute to the accumulation of misfolded proteins in age-related conditions as well. For HGPS accumulation of misfolded proteins, and damaged organelles such as mitochondria have been reported (Viteri et al., 2010; Vidak et al., 2023), as well as in WS (Maity et al., 2018; Fang et al., 2019) and CS (Scheibye-Knudsen et al., 2012; Alupei et al., 2018; Qiang et al., 2021). In HGPS this is further exacerbated by the primary cause of the disease, i.e., accumulation of progerin at nuclear periphery that activates the UPR (Vidak et al., 2023). On the other hand, in physiological aging and in WS and CS accumulation of garbage is partly also caused by reduced garbage disposal pathways through either unfolded protein response (UPR), chaperone mediated autophagy, proteosomal activity or macroautophagy (Lopez-Otin et al., 2023). Studies in CS and WS revealed impaired removal of damaged mitochondria through mitophagy highlighting the enrollment of both CSB and WRN in this process. A plausible mechanism for defective mitophagy in progeroid syndromes, CS, WS, XP and AT, appears to lie in defective DNA damage repair associated with persistent PARP1 activation depleting NAD+ cell reservoirs. This in turn leads to inhibition of NAD-dependent deacetylase Sirt, its downstream target PGC1α affecting the key protein involved in the process of mitophagy, uncoupling protein 2 (UCP2) (Fang et al., 2014; Fang et al., 2019). Thus, this signalling cascade provides key mechanic insights that relate to NAD+ depletion known to play a fundamental role in the biology of aging (Verdin, 2015). In HGPS, autophagy (Marino et al., 2008), UPR (Vidak et al., 2023) and mitophagy (Monterrubio-Ledezma et al., 2023) are apparently not compromised but even increased likely in an attempt to compensate for massive progerin accumulation. Consistent with this notion Vidak and colleagues have shown that in HGPS UPR machinery of the endoplasmic reticulum senses nucleoplasmic progerin aggregates through clustered Sun proteins a mechanism that awaits to be tested in physiological age-related conditions (Vidak et al., 2023).

### Antagonistic aging hallmarks

Finally, in all progeroid syndromes, similar to physiological aging, increased cellular damage will result in accumulation of cell cycle arrested senescent cells, mitochondrial dysfunction and deregulated nutrient sensing as an initial antagonistic response to counteract damage that becomes eventually detrimental (Table 1). Accordingly nutrient sensing pathways involving somatotrophic growth hormone (GH)-insulin growth factor (IGF) axis that promotes growth via mTOR signalling pathway decreases in aging (Lopez-Otin et al., 2013) and progeroid disorders WS (Sawada et al., 2023), CS (Schumacher et al., 2008; Vessoni et al., 2016) and HGPS (Marino et al., 2008) presumably in an attempt to decrease damage through cessation of growth. In HGPS this may not apply to muscle progenitor cells in which conversely elevation of mTOR was observed (Kawakami et al., 2019).

Mitochondrial deficiency that may be beneficial in its mild forms due to mitohormesis peaks in accumulation of damaged mitochondria associated with ATP reduction and elevated reactive oxygen species (ROS) in aging (Lopez-Otin et al., 2013) and similarly in progeroid syndromes WS (Fang et al., 2019), CS (Scheibye-Knudsen et al., 2012) and HGPS (Viteri et al., 2010; Rivera-Torres et al., 2013; Mateos et al., 2015). To some extent this may be attributed to direct effects of affected proteins in progeriod disorders in such that CSB localization to mitochondria may stimulate mitochondrial DNA (mtDNA) damage repair, WRN ability to stimulate transcription of NAD + biosynthethic enzyme, nicotinamide nucleotide adenylyltransferase 1 (NMNAT1) (Fang et al., 2019) and in HGPS deteriorating effects of progerin nucleoplasamic aggregates activating UPR (Vidak et al., 2023).

Accumulation of damage in chronological aging as well as accelerated damage in progeroid syndromes mainly contributes to the development of cellular senescence, which is a permanent arrest in cell cycle. Senescent cells gradually increase in numbers during chronological aging with variable extent depending on tissue origin and disease progression such as in the case of atherosclerosis (Childs et al., 2016; Lopez-Otin et al., 2023). Senescent cell burden is particularly high in aortic, liver, and kidney tissues of naturally aged and premature aged Ercc1-/Δ mice (Yousefzadeh et al., 2020). Many studies have shown accumulation of senescent cells in HGPS (e.g., (Cao et al., 2011a; Osorio et al., 2011) with reports also in WS and CS (Wang et al., 2020; Bramwell and Harries, 2023). Particularly affected by cellular senescence are endothelial cells (EC) and vascular smooth muscle cells (VSMCs) in HGPS (Osmanagic-Myers et al., 2019; Atchison et al., 2020; Benedicto et al., 2021; Manakanatas et al., 2022; Xu et al., 2022) and adipose tissue derived stem cells in WS (Sawada et al., 2023). This is consistent with strong impact of these diseases in cardiovascular and adipose tissue. Thus, selected progeroid disorders may serve as good models to study the effects of senescent cell on particular disease states.

### Integrative aging hallmarks

Accumulation of senescent cells is detrimental to micro- and macroenvironment through development of senescence-associated secretory phenotype (SASP) promoting inflammation as reviewed in (Coppe et al., 2010). This altered intercellular communication together with many other factors contributes to chronic inflammation associated with stem cell exhaustion and dysbiosis. These are parts of integrative response to damage that eventually leads to loss of tissue homeostasis in aging. Chronic inflammation in adipose tissue is shown to significantly reduce the adipogenic potential of adipose-derived stem cells in WS that may explain strong subcutaneous fat loss observed in this disease that may also be relevant to loss of fat tissue in physiological aging (Sawada et al., 2023). In HGPS selectively introducing aged progerin expressing endothelial cells in mice results in severely altered communication of these cells with their environment inducing myofibroblast switch in surrounding fibroblasts and promoting fibrosis of cardiovascular tissues (Osmanagic-Myers et al., 2019). Furthermore, chronic inflammation is observed involving increased immune cell infiltration in different tissues associated with elevation of SASP factors and pronounced plasma secretion of senescence-associated microRNAs (SA-miRs). Inhibition of one of these SA-miRs, miR 34a-5p was shown to alleviate cellular senescence phenotype, an approach that may be beneficial in physiological aging as well (Manakanatas et al., 2022). In contrast to HGPS, in CS, chronic inflammation, residing mainly within non-vascular neural tissues involving glial cell activation, appears to exacerbate the function of the surrounding vasculature disrupting the blood-brain barrier (Kajitani et al., 2021).

**FIGURE 3:**
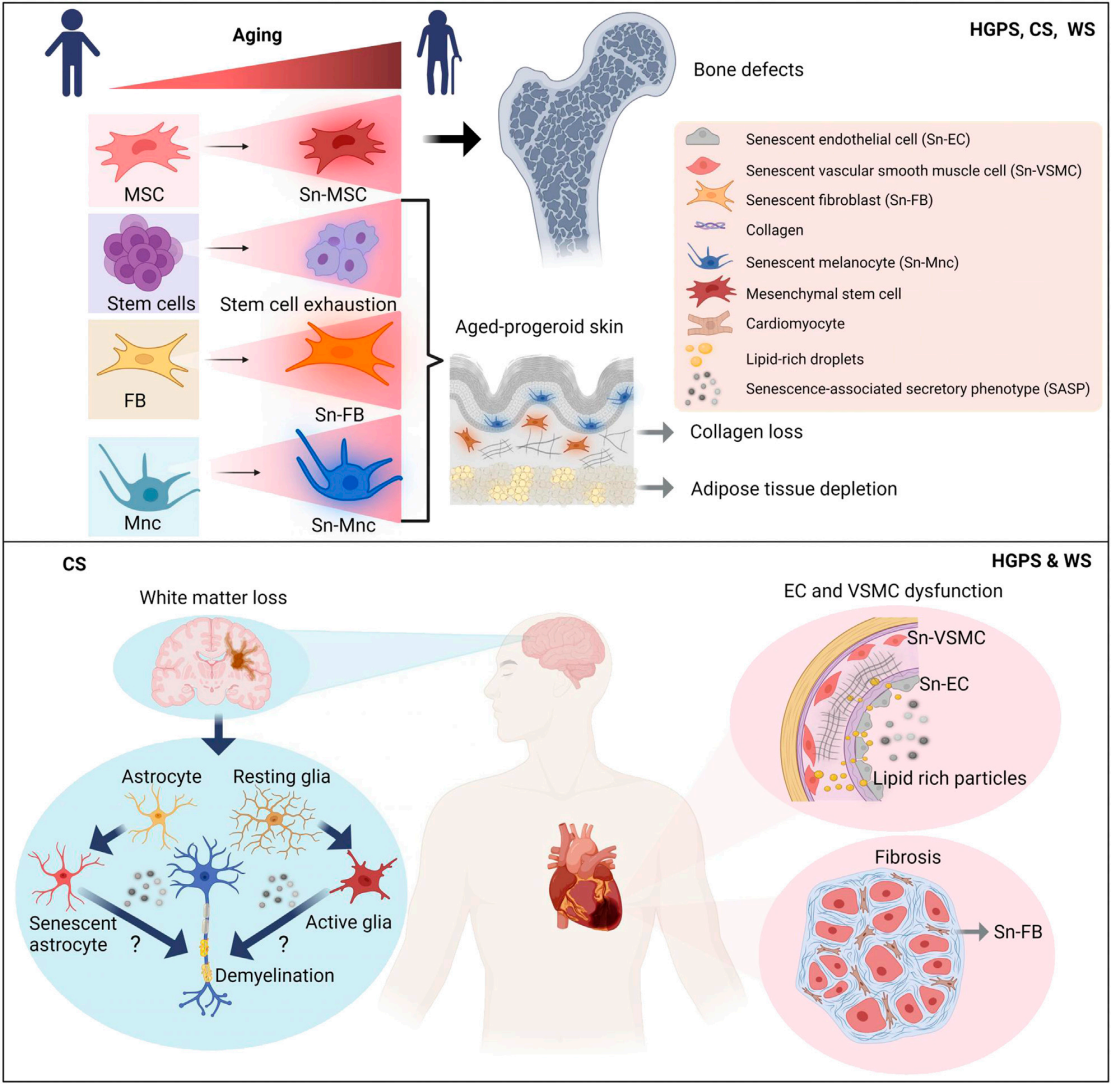
Models depicting accelerated tissue aging in progeriod disorders HGPS, WS and CS according to prevalent disease pathologies. Whereas accelerated cardiovascular aging is a prevalent characteristic in HGPS and WS, accelerated neural aging is predominant feature in CS. Skin and bone aging is a common characteristic for all three progeriod disorders. Simplified mechanistic insights into key aging hallmarks of respective disease pathologies are depicted. Sn, senescent; MSC, mesenchymal stem cell; FB, fibroblasts; Mnc, melanocyte; EC, endothelial cell; VSMC, vascular smooth muscle cell; SASP, senescence-associated secretory phenotype. Figure created with Biorender.com.

Altered intercellular communication, chronic inflammation and other age-related changes in the microenvironment eventually lead to stem cells exhaustion associated with impaired tissue regeneration and its reduced ability for repair after injury in aging (Brunet et al., 2023). Similar to physiological aging in progeroid syndromes reduced differentiation potential of stem cells, particularly mesenchymal stem cells in HGPS (Scaffidi and Misteli, 2008; Choi et al., 2018), WS (Fang et al., 2019; Sawada et al., 2023), CS (Wang et al., 2020), muscle derived stem cell progenitors in HGPS (Kawakami et al., 2019) and depletion of stem cell pool in the epidermis in HGPS (Rosengardten et al., 2011) is reported. The latter together with accumulation of different senescent cell populations may contribute to the characteristic skin and bone phenotype in these diseases as depicted in more detail in Figure 3 and in the next chapter.

Lastly introduced hallmark of aging is dysbiosis that characterizes the altered host-gut microbiota bidirectional communication associated with the reduction of bacterial diversity in aging (Lopez-Otin et al., 2023). Dysbiosis was also found in HGPS and NGPS patients and corresponding mouse models. In HGPS and NGPS models, Barcena and colleagues could show enhanced healthspan and lifespan upon fecal microbiota transplantation from wild-type animals that should open new avenues in treatment and our understanding of age-related disease conditions (Barcena et al., 2019). Altogether as demonstrated by interconnectedness of exemplified aging hallmarks it is not surprising that exaggerated primary damage observed in progeroid syndromes will further exacerbate antagonistic and integrative hallmarks. Consistent with this, almost all 12 hallmarks were reported for selected progeroid syndromes, HGPS, WS and CS (Table 1).

## Progeroid syndromes: Accelerated aging models of characteristic diseases

Due to their segmental nature, clinical features in HGPS, WS and CS, appear to resemble accelerated forms of physiological aging more or less accurately only for certain tissues subsets (Burtner and Kennedy, 2010). Here we want to elaborate on lessons so far gained from studying these progeroid syndromes particularly relevant to molecular mechanisms underlying characteristic age-dependent deterioration of specific tissues. In this respect HGPS and WS are particularly interesting since similar clinical features such as accelerated cardiovascular disease and atherosclerosis are observed in both diseases despite apparently different molecular functions of affected proteins. This phenomenon may at least partly be explained by interconnectedness of aging hallmarks as elaborated above. On the other hand, CS and XP as opposed to WS, despite similar engagement in DNA damage repair pathways, exhibit fundamental differences in phenotypes developing neurological abnormalities associated with intellectual disability in affected patients. Here, it is presumably the crucial function of CS and XP proteins in specific type of DNA-repair, NER, as described in detail below that may be of key importance for neural tissues. In this part we will describe the most prevalent disease characteristics in selected progeroid disorders in human patients but also mouse and cell models and at relevant parts provide mechanistic links to specific age-related disease pathologies.

### Cardiovascular diseases in HGPS and WS

One of the most pronounced clinical features in HGPS is severe cardiovascular disease characterized by preserved ejection fraction and significantly increased E/E' ratio indicating diastolic dysfunction (Prakash et al., 2018). Diastolic dysfunction together with many other functional changes of the cardiovascular system strongly resemble CVD in aging population (Chang et al., 2014; Hamczyk et al., 2018a). Moreover, severe atherosclerosis develops early in the childhood of HGPS patients with characteristics of intimal thickening, cholesterol crystals and necrotic core regions in such resembling atherosclerosis features of aging population (Olive et al., 2010). Similar to HGPS, in WS patients, age-related atherosclerosis, myocardial infarction but also arteriosclerosis obliterans are reported suggesting a common “aging” denominator as the upstream cause for such alterations as depicted in Figure 3 (Okabe et al., 2012; Oshima et al., 2017; Kato and Maezawa, 2022). However, in contrast to HGPS patients that show no significant changes in lipid profile with merely HDL-cholesterol levels decreasing with age (Gordon et al., 2005), WS patients develop severe hyperlipidemia associated with T2DM (Okabe et al., 2012) indicating different initial triggers of CVD in these two progeroid disorders.

In HGPS a whole plethothora of research in different HGPS mouse models has provided more clarity for the cellular basis of accelerated cardiovascular aging pathology as elaborated in (Benedicto et al., 2021). Severe vascular smooth muscle cell (VSMC) depletion and increased propensity to development of atherosclerosis in VSMC conditional HGPS mouse models indicates key importance of this cell type in CVD progression (Hamczyk et al., 2018b). On the other hand, endothelial dysfunction in ubiquitous HGPS mouse model Lmna^G609/G609G^ (Del Campo et al., 2020; Sun et al., 2020) and diastolic dysfunction associated with strong profibrotic changes of aging vasculature in endothelial conditional HGPS mouse models (Prog-Tg) highlights the importance of the endothelial system in the disease progression as well. Moreover, compromised alignment of progerin-expressing vasculature to blood flow showing reduced nuclei elongations (Osmanagic-Myers et al., 2019) is very much reminiscent of defective endothelial mechanoresponse observed during physiological aging (Collins and Tzima, 2011; Tian et al., 2022). A detailed comparison of the above findings between HGPS and WS is still hard to be made due to relatively mild pathology reported for *Wrn* ^*−/−*^ mouse model system (Lebel and Leder, 1998). Significant pathology, however, with no reports on cardiovascular phenotype is only found after additional deletion of telomerase RNA component (Terc^−/−^Wrn^−/−^mice) (Chang et al., 2004; Chang, 2005). Thus, future research on CVD in Terc^−/−^Wrn^−/−^mice and generation of atheroprone Terc^−/−^Wrn^−/−^mice may reveal additional insights into CVD disease pathology and affected cell types in WS.

On the mechanistic level much of the new findings regarding CVD in progeriod syndromes came from the research on HGPS patient derived iPSCs differentiated to VSMC and EC lineages, respectively. Accordingly, increased DNA damage signals, reduced telomeres, inflammation, and abundant cellular senescence in dysfunctional VSMCs as well as ECs were reported in HGPS (Atchison et al., 2020; Xu et al., 2022). Using endothelial-specific progeria mice, accelerating aging in ECs, was shown to be accompanied by strong paracrine effects on surrounding tissues leading to global spread of paracrine senescence and inflammation (Sun et al., 2020; Manakanatas et al., 2022). This highlights the massive extent of tissue damage that is exerted by introducing progeroid features in just one particular cell type, endothelial cells. Similar scenario may be expected for WS, since knockdown of *WRN* elevates inflammatory phenotype of endothelial cells (Laarmann et al., 2019). For HGPS mouse models, adopted endothelium-targeted Sirt7 therapy and usage of microRNA agents, shown to ameliorate aging features in progerin-expressing ECs, may prove to be successful in treatment of CVD in progeroid diseases and those associated with physiological aging as well (Sun et al., 2020; Manakanatas et al., 2022). Altogether these findings demonstrate the power of using progeroid particularly tissue specific mouse models for aging research.

### Non-cardiovascular diseases in HGPS and WS

Loss of subcutaneous fat, scleroderma-like phenotype but also osteopenia and osteolysis for HGPS and osteoporosis for WS with no effects in neural tissue for HGPS implicates pronounced premature aging of the skin and bone in these progeroid syndromes as depicted in Figure 3 (Chang et al., 2004; Merideth et al., 2008; Osorio et al., 2011; Oshima et al., 2017). Much of the research regarding these defects has indicated stem cell depletion, similarly observed in physiological aging (Brunet et al., 2023), as un underlying cause for these distinct phenotypes. For instance, increased cellular senescence associated with depletion of adult stem cells in the epidermis and adipose tissue was shown to accompany the skin phenotype in HGPS and WS, respectively (Rosengardten et al., 2011; Sawada et al., 2023). On the other hand, the bone phenotype in HGPS was demonstrated to be associated with loss of mesenchymal stem cells (MSCs) associated with their reduced potential to differentiate to osteoblast lineages (Scaffidi and Misteli, 2008; Choi et al., 2018). Regarding latter, recent advances in transcriptomic and Hi-C genomic analysis techniques appeared to have shed some light on MSC involvement in bone phenotype. Martin and colleagues could show that HGPS cells show a general misregulation and shifts in genome compartmentalization that particularly appear to coincide with expression changes of key mesenchymal lineages genes (San Martin et al., 2022). Altogether, these findings may provide important new insight into how changes in the epigenetic landscape occur during the aging process and how these in turn contribute to stem cell depletion and loss of cell identity as well.

### Neurodegenerative disease in CS

For CS (Karikkineth et al., 2017; Han et al., 2023) and many types of XP (Niedernhofer et al., 2011) the most severe pathology seems to be in the neuronal system resembling to some extent sporadic age-dependent neurodegenerative diseases. Similarly, to age-dependent dementias and sporadic late onset Alzheimer’s disease (LOAD) (Bouhrara et al., 2018; Lin et al., 2020), CS is characterized by neuronal loss with severe demyelination, however with so far no detectable amyloid-beta accumulation and no hyperphosphorylated tau (Woody et al., 1991; Han et al., 2023). Demyelination process implicates additional defects in oligodendrocytes of the central- and Schwann cells of the peripheral nervous system in CS disease progression. Thus, we may gain important mechanistic insights from studying CS animal models for underlying causes in CS that may be valuable for age-dependent neurodegenerative diseases.

However, research on CS, has been initially hampered by lack of adequate CS rodent models resembling human pathology. Hence, the first generated CS models, Csb−/− and Csa−/−, displayed only mild phenotypes with no obvious changes in myelin sheets typical for CS patients (van der Horst et al., 1997; Jaarsma et al., 2011). In mice with additional knockout of Xpa−/−, another protein involved in NER, *Csa* ^*−/−*^ *Xpa* ^*−/−*^, severe neurological defects with hindlimb paralysis and dystonia were observed including progeroid features such as kyphosis and lipodystrophy. However, the analysis of these mice was limited, due to their premature death at 28 days which was extended to∼16 weeks in mice on soft diet (Brace et al., 2013). Finally, Csb−/− mice with conditional knockout of Xpa−/− in postnatal neurons appeared to resemble very much CS patient pathology showing typical demyelination and progressive neuronal degeneration (Jaarsma et al., 2011; Kajitani et al., 2021). On the cellular level Mac2-positive microglia with elevated levels of glial fibrillary acidic protein indicated increased microglia-activation and neuronal injury (Jaarsma et al., 2011). For both, neurons and astrocytes, increased levels of activated p53 accompanied by elevated apoptosis suggested activation of p53-apoptotic- or senescence pathway that likely was the cause for substantial neural loss (Jaarsma et al., 2011). According to the current state of the research, abnormal activation of microglia and astrocytes emerge as key contributors to neuoroinflammatory response of aging brain tissue indicating high resemblance of CS to age-dependent neurodegenerative diseases in elderly and LOAD (Hou et al., 2019) as proposed in Figure 3.

The question that arises at this point is why accelerated aging of the neural tissue appears to be the prevalent characteristic in CS. One of the plausible explanations for this phenomenon might lie in the huge demand of neural tissue for oxygen causing increased generation of reactive oxygen species (ROS) and accumulation of DNA damage (Madabhushi et al., 2014). As implicated previously (Niedernhofer, 2008), in neural tissue TC-NER may be the essential way to repair such accumulated DNA-damage, providing explanation why in CS, harboring defective TC-NER, specifically premature neural aging is observed. Consistent with accumulated unrepaired DNA damage in neural tissue, increased senescent neural cell burden is reported in previous findings showing increased levels of p53-positive cells in neural tissues and cell cultures derived from CS patients (Jaarsma et al., 2011; Bramwell and Harries, 2023). In analogy to this, LOAD, for which the highest risk factor is aging, is characterized by accumulation of ROS and accumulated DNA damage accompanied by increased senescent cell burden suggesting common triggers in age-dependent neurodegenerative diseases and CS (Lau et al., 2023).

Emerging new technologies provided a deeper mechanistic insight into underlying causes of CS-pathologies that may be applicable to physiological aging as well. For instance, transcriptome analyses of several Csb−/− cell lines have implicated Csb in chromatin remodeling able to affect active histone marks in gene promoter regions. In such a way, Csb was shown to suppress the key negative regulator of neuron-specific growth, Necdin. Accordingly, *NDN* depletion, in CSB−/− neural cell lines and Csb-mouse models, is shown to partially rescue motor neuron deficits (Liang et al., 2023). Thus, this finding highlights the importance of exploring direct but also indirect targets in regulation of CS pathologies that may help solve puzzles in complex pathways involved in age-related neurodegeneration. Moreover, side-by-side comparison of premature and physiological aging, may help identify the pattern of long-term global changes involved in complex neuronal pathologies of CS and elderly. Accordingly, Lim and colleagues have used a new PhaseDel technology in single-cell whole genome sequencing of a large number of single neurons from aged and progeroid individuals (CS, Xeroderma pigmentosum and Ataxia telangiectasia) to demonstrate accumulation of somatic mutations particularly in DNA-repair genes as potential underlying cause of neurodegenerative disorders in aged individuals (Kim et al., 2022).

### CS in non-neural tissues

It is not to exclude that in CS also non-neural tissues resemble to some extent accelerated forms of physiological aging. For instance, abnormal accumulation of subarachnoid arteries accompanied by subdural hematoma are typical diseases of the elderly that are often found in CS patients (Weigel et al., 2022). These may implicate common vascular changes in affected brain regions in CS and elderly. Atherosclerosis appears to be an atypical feature in CS patients (Hayashi et al., 2012) in contrast to HGPS and WS (Kato and Maezawa, 2022). Recent studies in CS mouse model, *Csa−/−Xpa−/−*, found no changes in isolated endothelial cells or aorta (Kajitani et al., 2021) suggesting rather non-cell autonomous effects (Table 1) as an underlying cause for brain vascular defects thereby favoring the hypothesis of neural-centric view of aging in CS (Figure 3).

Regarding bone tissue in CS it is difficult to discriminate intrinsic from extrinsic effects. One early CS case showing skeletal dysplasia (Cirillo Silengo et al., 1986), general dwarfism, no incidents of bone fractures and frequently observed osteoporosis are observed (Laugel, 2013). Some of these defects may be rooted in secondary causes such as increased joint contractures and reduced mobility in these patients (Laugel, 2013). On the other hand, reduced differentiation potential to osteoblastic lineages of mesenchymal stem cells obtained from CS-iPSCs pointed rather to intrinsic bone defects in CS (Wang et al., 2022) as outlined in Figure 3. Skin, adipose and renal tissue are affected in CS with typical accelerated aging features such as lipodystrophy, cutaneous photosensitivity (Figure 3), and nephoronic reduction shown also in different CS rodent models (Jaarsma et al., 2013; Laugel, 2013). However, to what extent on the cellular levels these non-neural features resemble physiological aging will require further investigation.

## Intervention strategies

Intervention strategies with therapeutic potential may be subdivided into those designed specifically for progeroid disorders and broad interventions mostly applicable for common age-related diseases as well. For HGPS, FDA has even approved in November 2020 a drug Lonafarnib (Zokinvy™) that significantly reduces the risk of mortality in HGPS patients (Dhillon, 2021). Lonafarnib is an orally active farnesyltransferase inhibitor that prevents farnesylation and accumulation of progerin and thus its toxic effects (Gordon et al., 2012). Furthermore, remarkable advances are observed in experimental interventional strategies applicable for particular progeroid disorder such as gene editing procedures in HGPS (Beyret et al., 2019; Santiago-Fernandez et al., 2019; Koblan et al., 2021; Whisenant et al., 2022) and in CS (Wang et al., 2022), morpholino oligos to target aberrant splicing in HGPS (Osorio et al., 2011) and antisense oligonucleotides (ASOs) that reduce the levels of progerin transcripts (Puttaraju et al., 2021). The latter oligo-based treatments that require no change in patient’s DNA, may pave ground to alternative treatment options of specific age-related conditions. This is exemplified by work of Auguado and colleagues, who reported on sequence-specific telomeric antisense oligonucleotides (tASO) that were able to prevent the DNA damage response at dysfunctional telomeres. Thereby, tASOs reduced cellular senescence, improved skin homeostasis and life span of HGPS mouse models *in vivo* (Aguado et al., 2019). Such approaches may be effective in treatment of other progeroid disorders but may also represent first attempts in targeting fundamental causes of “aging” that my prove efficient in treatment of multiple age-related disorders as proposed in “geroscience hypothesis” (Sierra and Kohanski, 2017).

Very effective broad experimental interventions in age-related diseases and in progeroid disorders proved to be treatments affecting the so called “waste generation”, “waste disposal pathways” (aging hallmark: loss of proteostasis and disabled macroautophagy) and agents reducing cellular senescence (aging hallmark: cellular senescence). In such way, treatments with chemical chaperons, that reduce the burden of misfolded proteins and ROS (waste), have been highly beneficial intervention strategies in CS (Alupei et al., 2018; Qiang et al., 2021), and HGPS (Hamczyk et al., 2019) as well as those affecting the waste disposal pathway such as rapamycin, spermidine or NAD+ precursors. Rapamycin was shown to restore the mitochondrial function in CS (Scheibye-Knudsen et al., 2012), and to reduce misfolded proteins, likely also progerin at the nuclear membrane in HGPS (Vidak et al., 2023) thereby reversing the senescent phenotype of HGPS fibroblasts (Cao et al., 2011b). Its beneficial effects have been mainly attributed to the increase in autophagy. Mechanistically, rapamycin exerts inhibitory effects on mTORC1, a key regulator of autophagy, proteostasis, inflammation and senescence phenotype (Linke et al., 2017; Lopez-Otin et al., 2023).

Similarly, autophagy inducer spermidine was proven to be highly beneficial in treatment of WS (Yang et al., 2020), HGPS-like Zmpste24−/− mice (Ao et al., 2019) with cardioprotective effects in aged rodents as well (Eisenberg et al., 2016). Furthermore, in WS, NAD+ precursors were shown to ameliorate accelerated aging features including stem cell dysfunction through boosting NAD+ and mitophagy (Fang et al., 2019). Finally, selective elimination of senescent cells termed senolysis, was demonstrated to be effective in delaying aging-associated diseases first for premature aging mouse model BubR1 (Baker et al., 2011), then in physiologically aged mouse model (Baker et al., 2016) and recently specifically in HGPS mouse model as well (Hambright et al., 2023).

Finally, experimental interventions involving cellular reprogramming using short-term cyclic expression of Yamanaka factors Oct4, Sox2, Klf4 and c-Myc that target the aging hallmark “epigenetic alterations” were proven highly effective in ameliorating the age-dependent phenotypes and improving life span in progeroid HGPS mouse models (Ocampo et al., 2016; Alle et al., 2022). This appeared to have paved the ground to a slightly modified strategy that omits c-Myc. The latter employs cyclic expression of OSK in retinal ganglions which was shown to promote axon regeneration (Lu et al., 2020). Such experimental approach harbors a high therapeutic potential for treatment of glaucoma, which is one of the prevalent diseases in the elderly. Altogether, from these findings it is evident that broad interventions share many common mechanistic links in disease pathologies of accelerated and physiological aging highlighting progeroid cell and rodent models as valuable tools in developing future therapeutic intervention strategies to combat age-related diseases.

## Conclusion

As outlined in this review progeroid syndromes share common hallmarks with those observed in physiological aging and reflect more or less accurately accelerated aging of particular tissues. Much of the evidence causally linking those hallmarks to physiological aging came from studies in premature aging animal models that exhibit those hallmarks in exaggerated fashion as exemplified on the pronounced genomic instability in WS. The availability of different progeroid animal models, particularly in HGPS field, has very much pushed the research field of aging forward allowing us to gain important molecular insights into basic aging mechanisms and draw conclusions relevant for age-related diseases. Particularly, generation of tissue-specific accelerated aging mouse models enables selective introduction of specific aging cell-type into organism as a whole in order to decipher in a cell-type specific manner mechanisms underlying distinct disease pathologies of aging. In sum, advances in technologies combined with future studies in progeroid syndromes are expected to reveal further mechanistic insights into fundamental processes driving age-related pathologies.

## Figures and Tables

**Table:** 

**AP**	apurinic
**APS**	atypical progeria syndromes
**AT**	ataxia telangiectasia syndrome
**ATM**	ataxia telangiectasia mutated
**BER**	base excision repair
**BS**	Bloom syndrome
**CS**	Cockayne syndrome
**CVD**	cardiovascular disease
**DC**	dyskeratosis congenita
**DNA-PK**	DNA dependent protein kinase
**DDR**	DNA damage repair
**DSB**	double strand break
**DSBR**	double-strand break repair
**EC**	endothelial cell
**ERCC**	excision repair cross complementation
**FA**	Fanconi anemia
**GFAP**	glial fibrillary acidic protein
**GG-NER**	global genome nucleotide excision DNA repair
**GH**	growth hormone
**HGPS**	Hutchinson-Gilford progeria syndrome
**HHS**	Hoyeraal-Hreidarsson syndrome
**HP1a**	heterochromatin protein 1 alpha
**HR**	homologous replication/recombination
**IGF**	insulin growth factor
**ICLR**	interstrand DNA crosslink repair
**iPSCs**	induced pluripotent stem cells
**LAD**	lamina-associated domain
**LINC**	linker of nucleoskeleton and cytoskeleton complex
**LOAD**	late onset Alzheimer’s disease
**MADA/MADB**	mandibuloacral dysplasia type A and B
**MSCs**	mesenchymal stem cells
**mtDNA**	mitochondrial DNA
**mTOR**	mammalian target of rapamycin
**NAD+**	nicotinamide adenine dinucleotide
**NBS**	Nijmegen breakage syndrome
**NGPS**	Nestor-Guillermo progeria syndrome
**NHEJ**	non-homologous end joining
**NMNAT1**	nicotinamide nucleotide adenylyltransferase 1
**PARP1**	poly (adenosine-diphosphate-ribose) polymerase 1
**PGC1α**	peroxisome proliferator-activated receptor gamma coactivator 1-alpha
**RCM**	relocation of chromatin modifiers
**RD**	restrictive dermopathy
**ROS**	reactive oxygen species
**RTS**	Rothmund-Thomson syndrome
**SASP**	senescence-associated secretory phenotype
**SA-miRs**	senescence-associated microRNAs
**SSB**	single-strand DNA break
**ssDNA**	single stranded DNA
**tASO**	telomeric antisense oligonucleotides
**TC-NER**	transcription coupled nucleotide excision DNA repair
**T2DM**	type 2 diabetes mellitus
**TET**	Ten-eleven translocation enzyme
**TTD**	trichothiodystrophy
**UCP2**	uncoupling protein 2
**UPR**	unfolded protein response
**VSMC**	vascular smooth muscle cell
**WRN**	Werner helicase
**WS**	Werner syndrome
**XP**	xeroderma pigmentosum
